# Impact of individualized and structured aerobic exercise on clinical outcomes in pediatric congenital heart diseases with post-surgical rehabilitation: a meta-analysis

**DOI:** 10.3389/fsurg.2025.1622547

**Published:** 2025-11-26

**Authors:** Ayoola Awosika, Tirath Patel, Shree Rath, Fathimathul Henna, Umama Alam, Aizaz Ali, Mayowa Jeremiah Adeniyi

**Affiliations:** 1 Department of Family Medicine, University of Illinois College of Medicine Peoria, Bloomington, IL, United States; 2 Department of Surgery, Houston Healthcare, Warner Robins, GA, United States; 3 Department of Surgery, All India Institute of Medical Sciences, Bhubaneswar, India; 4 Department of Surgery, Dubai Medical College for Girls, Dubai, United Arab Emirates; 5 Department of Surgery, Khyber Medical College, Peshawar, Pakistan; 6 Department of Physiology, Federal University of Health Sciences Otukpo, Benue, Nigeria

**Keywords:** congenital heart diseases, cardiac rehabilitation, exercise duration, meta-analysis, cardiac disease

## Abstract

**Background:**

Children with congenital heart defects (CHD) commonly experience decreased exercise capacity due to structural heart abnormalities, surgical interventions, and parental-and physician-imposed activity restrictions. This reduced activity can lead to physical deconditioning, impaired quality of life, and increased cardiovascular risk later in life. While exercise-based rehabilitation is highly recommended, significant knowledge gaps persist regarding the long-term impact of structured exercise on diverse CHD subtypes, optimal modalities, and standardized protocols for implementation. This meta-analysis assesses the effect of structured exercise rehabilitation programs on functional and health-related outcomes in children with CHD.

**Methods:**

A comprehensive search was done using PubMed/MEDLINE, Embase, and Web of Science until April 23, 2025, for randomized controlled trials (RCTs) and observational studies which compares exercise or cardiac rehabilitation with standard of care or no rehabilitation intervention in pediatric CHD patients. Key outcomes included changes in exercise duration, peak oxygen uptake (peak VO2), peak workload, heart rate, and other cardiopulmonary parameters. Data were analyzed and pooled using random-effects models, with heterogeneity evaluated via I^2^ statistics. Risk of bias (RoB) was assessed using RoB 2 for RCTs and ROBINS-I for observational studies, and evidence certainty was assessed using the GRADE approach.

**Results:**

Ten studies (5 RCTs, 5 observational) comprising of 378 patients were included. Exercise rehabilitation significantly elevated exercise duration [MD = 0.55, 95% CI: (0.01, 1.09); *p* = 0.04; *I*^2^ = 0%]. No significant advancement was seen in peak VO2 [MD = 1.14, 95% CI: (−1.07, 3.34); *p* = 0.31; *I*^2^ = 69%], peak workload, heart rate, or other cardiopulmonary parameters. Heterogeneity was high for several outcomes, especially peak workload and VO2, which was settled in sensitivity analyses for specific subgroups. Evidence certainty was moderate due to heterogeneity and study limitations.

**Conclusion:**

Exercise rehabilitation moderately enhances exercise duration in pediatric CHD patients but does not notably enhance most cardiopulmonary parameters. High heterogeneity reflects outcomes variability by CHD subtype and intervention protocol. Standardized, multicenter trials are required to improve and optimise exercise prescriptions and evaluate long-term benefits.

## 1 Introduction

A congenital heart defect (CHD), also referred to as a congenital heart anomaly, congenital cardiovascular malformation, or congenital heart disease, is a structural abnormality of the heart or major blood vessels that is present at birth (1). CHD poses a significant challenge in pediatric healthcare, substantially contributing to infant morbidity and mortality on a global scale (2). In 2015, congenital heart disease accounted for approximately 303,300 deaths, marking a decline from 366,000 deaths reported in 1990 (3).

Patients with congenital heart diseases (CHD) typically exhibit several physiological complications, including reduced aerobic capacity, exertional breathlessness, cardiovascular and peripheral muscle deconditioning, as well as muscle weakness and atrophy (4). Within the context of this condition, exercise intolerance and fatigue are widely acknowledged as significant consequences of CHD (5). It is well established that sedentary lifestyles are linked to a higher risk of morbidities, including obesity, metabolic disorders, and cardiorespiratory risk factors later in adulthood. Promoting a more active lifestyle, along with implementing (6). Alarmingly, children with CHD have been shown to engage in lower levels of daily physical activity (7) and exhibit diminished exercise capacity, which has been linked to a lower health-related quality of life (8).

Current public health recommendations advise at least 60 min of moderate-to-vigorous physical activity each day for children and adolescents (9). Enhancing physical activity can boost cardiopulmonary fitness, and improved cardiopulmonary fitness, in turn, may encourage greater physical activity (10). Exercise training programs, whether home-based or facility-based, may enhance VO2 levels in patients with CHD (11) and may also enhance health-related quality of life (HRQOL) in overweight and obese children (12) In 2013, the American Heart Association endorsed physical activity for children and adults with congenital heart defects. These recommendations were largely adapted from those for healthy individuals, as specific guidelines tailored to this patient group are still absent. It was further concluded that, aside from patients with severe rhythm disorders, there is no evidence to support exercise restrictions. Although no specific advice was provided regarding types of exercise or sports, a general reduction in sedentary behavior was encouraged (13).

Despite the growing recognition of the benefits of physical activity in children with congenital heart defects (CHD), there remains a lack of consolidated evidence regarding the effectiveness of structured exercise rehabilitation programs in this vulnerable population. Individual studies have reported varying degrees of improvement in cardiopulmonary fitness, exercise capacity, and health-related quality of life (HRQOL), yet discrepancies in study design, intervention types, and outcome measures have limited the generalizability of findings (5, 7, 8). Moreover, there is currently no standardized guideline specifically tailored for exercise rehabilitation in pediatric CHD patients. Therefore, a comprehensive meta-analysis is needed to systematically evaluate the impact of exercise-based rehabilitation programs on functional and health-related outcomes in children with CHD. By synthesizing available data, this study aims to provide stronger evidence to inform clinical practice, promote physical activity interventions, and ultimately enhance the long-term health and quality of life of children living with congenital heart defects.

## 2 Methods

### 2.1 study design and protocol registration

**Figure 1:**
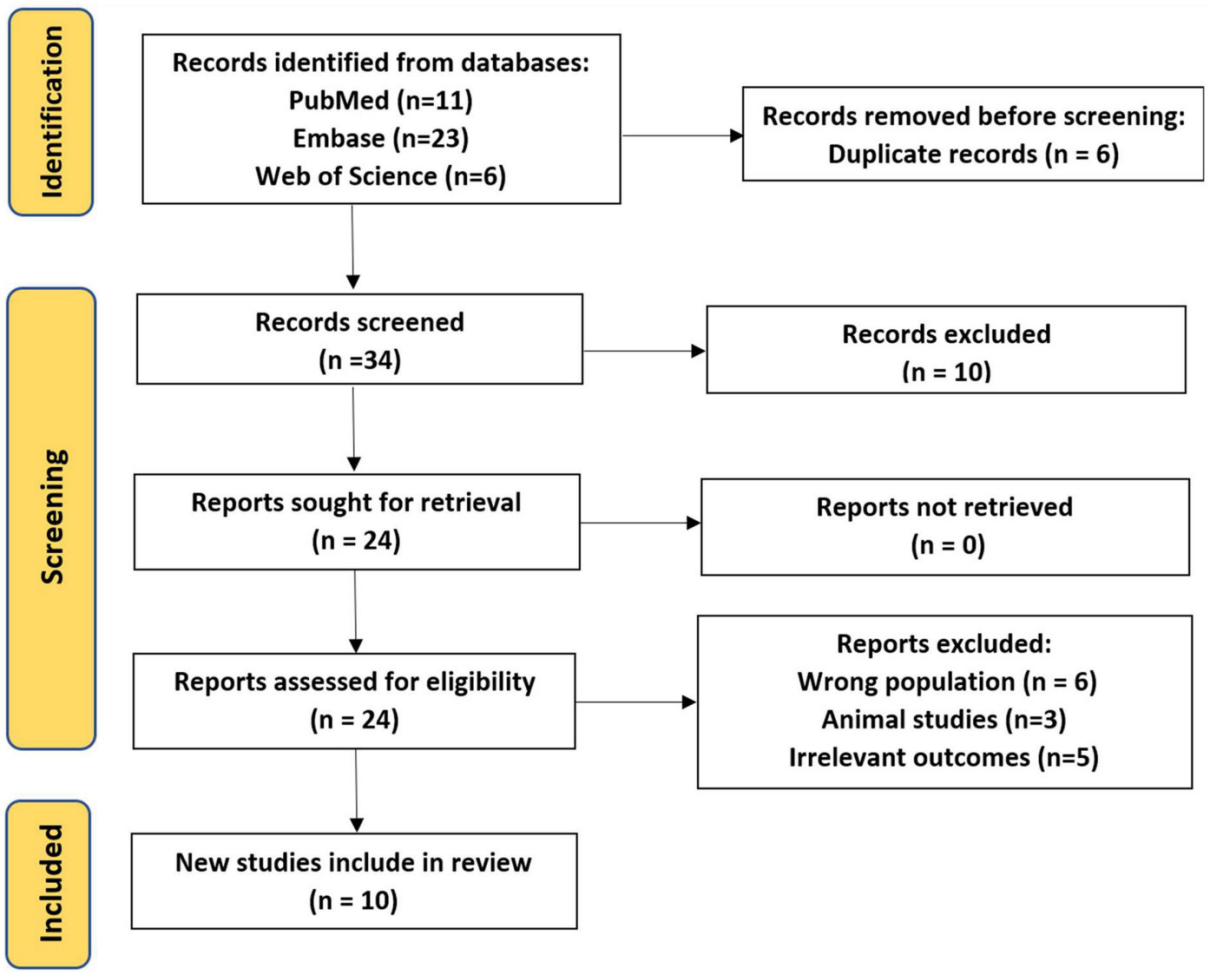
PRISMA flow diagram illustrating the study selection process for inclusion in this meta-analysis.

The meta-analysis was conducted according to the guidelines provided by the Cochrane Handbook for Systematic Reviews of Interventions and reported according to the preferred Reporting items for systematic review and meta-analysis (PRISMA) statement (14). (Figure 1).

### 2.2 data resources and search strategy

We systematically searched PubMed/MEDLINE, Embase, and Web of Science from their inception until 23 April 2025 to identify studies comparing exercise or cardiac rehabilitation with standard of care or no rehabilitation intervention in pediatric patients with CHD. A combination of the following Medical Subject Headings (MeSH) and keywords were used: “exercise therapy,” “cardiac rehabilitation,” “heart defects, congenital,” and “long QT syndrome.” Bibliographies of all included articles were also searched to identify additional relevant studies. Only studies that were published in the English language were included. This review did not consider gray literature, such as dissertations and unpublished studies. The detailed search strategy is provided in Supplementary Table S1.

### 2.3 study selection and eligibility criteria

Articles retrieved from the systematic search were exported to Rayyan AI, where duplicates were screened and removed. Titles and abstracts of the remaining articles were reviewed independently by two reviewers (U.A. and F.H.). Full texts of potentially eligible articles were assessed based on predefined criteria. Any disagreements during the review process were resolved independently through discussion with a third reviewer (S.R). The eligibility criteria were as follows: (1) Population: patients diagnosed with CHD; (2) Intervention: exercise or cardiac rehabilitation; (3) Comparator: standard of care; (4) Study Designs: Randomized controlled trials (RCTs) and observational studies; and (5) Outcomes: Studies reporting at least one relevant outcome.

### 2.4 data extraction and outcomes

Two authors (S,R. and.U.A) conducted data extraction independently using a predetermined Microsoft Excel spreadsheet. Any conflicts during the extraction process were resolved by a third author (F.H). Data were extracted from the study text, tables, and figures, with raw values estimated from percentages where necessary.

The extracted data included baseline characteristics such as country, study design, sample size, age, sex, BMI, CHD type, NYHA functional class, oxygen saturation, prior surgeries, baseline VO2, QOL tool and medication/devices.

### 2.5 Quality assessment

The quality of the included studies was assessed using appropriate tools based on their study design. For RCTs, the Revised Cochrane Risk of Bias Tool for Randomized Trials (RoB 2) (SupplementaryFigure S1, S2) (15) was used, evaluating bias across five domains: randomization process, deviations from intended interventions, missing outcome data, measurement of outcomes, and selection of reported results. Each study's overall risk of bias was categorized as low, some concerns, or high risk.

For observational studies, the Risk of Bias in Non-Randomized Studies of Interventions (ROBINS-I) tool (16) was applied, assessing bias across seven domains: confounding, selection of participants, classification of interventions, deviations from intended interventions, missing data, measurement of outcomes, and selection of reported results. The overall risk of bias was classified as low, moderate, serious, or critical.

Two reviewers (F.H and S.R.) independently assessed the risk of bias, resolving disagreements through discussion, with a third reviewer (U.A) consulted if necessary. This systematic assessment ensured the reliability and validity of the included studies.

#### 2.5.1 Grade assessment

Grading of Recommendations, Assessment, Development, and Evaluation (GRADE) tool, specifically the GRADEpro Guideline Development Tool (17), was used by two independent authors (M.A.A. and U.A.) to rate the degree of certainty in the evidence in this meta-analysis, classifying it from high to very low (18). Any discrepancies were discussed and settled by agreement.

### 2.6 statistical analysis and sensitivity analysis

Review Manager 5.4 was used to perform statistical analysis. Treatment effects for binary outcomes were compared using a pooled risk ratio (RR) with 95% confidence intervals (CI), while continuous outcomes were analyzed using mean differences (MD) with 95% CI. The Cochran Q test and I^2^ statistics were used to assess heterogeneity, with *P*-values < 0.10 and *I*^2^ > 50% considered indicative of significant heterogeneity (19). The DerSimonian and Laird random-effects model was applied to all outcomes (20). A *p*-value of <0.05 indicates statistical significance for clinical endpoints. The stability of the pooled estimates was assessed through a leave-one-out analysis, where each study was sequentially removed, and the remaining dataset was re-analyzed to ensure that no single study unduly influenced the aggregated effect sizes.

## 3 Results

### 3.1 Search results

The search identified 40 records: 11 from PubMed, 23 from Embase, and 6 from Web of Science. After removing 6 duplicate records, 34 records remained for title and abstract screening. Following the screening process, 10 records were excluded, and 24 full-text articles were assessed for eligibility. Of these, 14 were excluded (6 due to wrong population, 3 animal studies, and 5 for irrelevant outcomes), resulting in 10 studies being included in the final review (5 RCTs and 5 observational studies).

### 3.2 Study characteristics

This meta-analysis comprises a total of 10 included studies that met the inclusion criteria, composed of 5 randomized controlled trials (RCTs), 3 controlled intervention studies, 1 prospective interventional study, and 1 pilot feasibility study. The publication dates of the studies ranged from 2000 to 2024. All 10 studies were included in both descriptive and statuses analyses. In total, 868 pediatric patients with congenital heart disease were recruited from all studies, consisting of various intervention types such as structured exercise training and cardiac rehabilitation. The sample sizes among each study approximated from 14 to 163 participants. Detailed baseline characteristics of the included studies are summarized separately (Table 1).

**Table 1:** Baseline characteristics of studies included in the meta-analysis, detailing study design, patient demographics, CHD types, interventions, and key clinical parameters.

Study ID	Country/Setting	Design	Sample Size	Duration of exercise intervention	Age (mean ± SD)	Sex (% male)	BMI/Height/Weight	CHD Type Breakdown	NYHA/Functional Class	Oxygen Saturation	Prior surgeries/interventions	Baseline VO2/6MWT/CPET	QoL tool & baseline scores	Medications/Devices
Hedlund et al. (21)	Sweden	Prospective interventional	30 Fontan, 25 controls	12 weeks	14.2 ± 3.2	53.3%	BMI: 18.3 ± 2.2	100% Fontan	N/R	Not reported	Fontan at median 2.4 yrs	VO2: 35.0 ± 5.1 mL/kg/min; 6MWT: 590.7 ± 65.5 m	PedsQL: 70.9 ± 9.9 (child), 65.1 ± 18.0 (parent)	3 pacemakers, most on aspirin or enalapril
Moalla et al. (22)	France/Tunisia	RCT	18	12 weeks	13.5 ± 1.0	N/R	N/R	ToF, TGA, ASD, Fontan	NYHA Class II–III	Not reported	Surgical correction; LVEF <40%	MVC: 101.6 ± 14.0 N·m	N/R	No pacemakers; on diuretics, ACE-I
Dulfer et al. (8)	Netherlands	RCT	91 (54 int., 37 ctrl.)	12 weeks	15.2 ± 3.	72.2%	N/R	50% Fontan, 50% ToF	N/R	N/R	All surgically repaired	Peak VO2: 82.4% predicted	TACQOL baseline provided	Not specified
Amedro et al. (23)	France	Multicenter RCT	142	12 weeks	17.4 ± 3.4	48%	BMI: 21.8 ± 3.6 (int.), 20.9 ± 4.0 (ctrl.)	Mixed CHD; VO2 max <80%	NYHA I–III	N/R	All repaired CHD	VO2 < 80% predicted	PedsQL self-reported: 71.6 ± 15.2 (int.)	4% with pacemaker; 28% on meds
Rhodes et al. (24)	USA	Controlled longitudinal	33 (15 rehab, 18 control)	12 weeks	12.5 ± 2.	∼60%	N/R	Complex CHD (mostly Fontan, ToF)	Functional limitations noted	Variable	≥1 open-heart surgery	VO2: ∼25 ml/kg/min; Work rate ∼63% predicted	CHQ used	1.8 meds/patient; 2.7 surgeries/patient
Callaghan et al. (25)	UK	RCT	163	16 weeks	Mean 8.4	61.3%	N/R	Mixed CHD; cyanotic/acyanotic	NYHA I–II	∼95%–98%	All post-op or repaired	Peak EST duration 4.5 ± 1.9 min (cyanotic group)	N/R	N/R
Jacobsen et al. (26)	USA	Pilot feasibility study	14	12 weeks	10 ± 1.15	57%	N/R	36% dominant left ventricle (Fontan)	N/R	Measured pre/post-activity	Fontan completion before enrollment	Shuttle Test; VO2max estimated via formula	Parent PedsQL: Physical function improved	No adverse events; high adherence
Klausen et al. (27)	Denmark	RCT	158	52 weeks	14.6 ± 1.3	58%	BMI: ∼21 (girls), ∼19.5 (boys)	Mixed complex CHD	N/R	N/R	Multiple surgeries; stratified by VO2	VO2: girls 37.5, boys 47.9 mL/kg/min	Generic QoL: ∼80/100	N/R
Fredriksen et al. (28)	Norway	Controlled intervention study	129	2 weeks	13 ± 1.73	∼50%	N/R	Various CHD types	N/R	N/R	All operated	Peak VO2: improved post training	Child Behavior Checklist: improved internalizing	N/R
Duppen et al. (29)	Netherlands	RCT	90	12 weeks	Mean 15	73%	Weight: 55 kg	52% ToF, 48% Fontan	NYHA Class II in 24%	O2 sat: 97%–98%	Multiple; Fontan at 3 yrs, ToF at 6 months	VO2 increased 5.0% post-exercise (only ToF significant)	HRQoL not primary focus	7% on meds; arrhythmia hx in 8%

**Table 2:** Other secondary pooled outcomes.

Outcome	Number of pooled studies	Effect size [95% confidence interval]	Overall effect *P* value	Heterogeneity (I2, *P* value)
Change in exercise (moderate/severe)	2	MD: 0.31 [−5.84, 6.47]	0.92	50%; *P* = 0.16
Change in peak respiratory rate	2	MD: 4.64 [−7.28, 16.57]	0.45	82%; *P* = 0.02
Change in peak systolic blood pressure	2	MD: 0.55 [−0.36, 1.46]	0.24	0%; *P* = 0.56
Change in VAT%	2	MD: 5.32 [−5.21, 15.68]	0.32	89%; *P* = 0.002
Change in peak VO2	2	MD: 4.98 [−6.31, 16.27]	0.39	91%; *P* = 0.001

### 3.3 Risk of bias assessment

The risk of bias assessment categorized Duppen 2015 (29), Dulfer 2014 (8), Amedro 2024 (23) and Callaghan 2021 (RCTs) (25) as low risk while Klausen 2016 (RCT) (27) had some concerns due to deviations from intended interventions, bias due to missing outcomes and measurement of outcomes. Hedlund 2017 (21), Jacobsen 2016 (26), Rhodes 2006 (24), Fredriksen 2000 (28) and Moalla 2012 (22) (observational studies) had moderate concerns at least in 1 domain. Supplementary Figures S1–S4 report the detailed bias assessment.

### 3.4 Meta analysis of primary endpoints

#### 3.4.1 Change in the exercise duration from baseline

**Figure 2:**
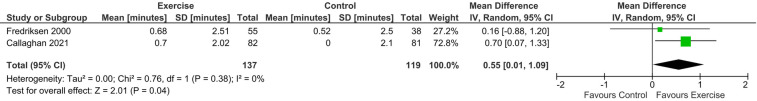
Forest plot showing the pooled mean difference (MD) for change in exercise duration (minutes) from baseline comparing exercise rehabilitation vs. standard care or no intervention.

2 studies comprising 256 patients (exercise = 137, control = 119) assessed change in the exercise duration over 12 and 16 weeks respectively from baseline. The pooled analysis revealed that the exercise group was associated with a significantly higher change in the exercise duration from baseline [MD = 0.55, 95% CI: (0.01, 1.09); *p* = 0.04]. No substantial heterogeneity was observed (*I*^2^ = 0%, *p* = 0.38), indicating high consistency across studies (Figure 2).

#### 3.4.2 Change in peak Vo2/mL/kg/min from baseline

**Figure 3:**
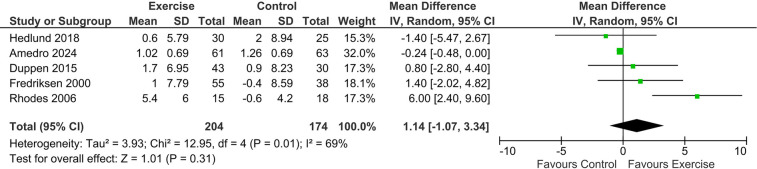
Forest plot presenting the pooled mean difference (MD) for change in peak VO₂ (mL/kg/min) from baseline comparing exercise rehabilitation vs. standard care or no intervention.

5 studies comprising 378 patients (exercise = 204, control = 174) assessed change in Peak VO2/mL/kg/min over 12 weeks from baseline. The results revealed no statistically significant difference between the 2 groups in terms of change in Peak VO2/mL/kg/min from baseline [MD = 1.14, 95% CI: (−1.07,3.34); *p* = 0.31]. Substantial higher heterogeneity was observed indicating variability across the studies (*I*^2^ = 69%, *p* = 0.01) (Figure 3).

### 3.5 Meta-analysis of secondary endpoints

#### 3.5.1 Change in exercise/ mod/ severe from baseline

2 studies comprising 209 patients (exercise = 110, control = 99) assessed change in exercise/ mod/ severe from baseline over 12 and 16 weeks respectively. The pooled analysis revealed no significant difference between exercise and control groups [MD = 0.31, 95% CI: (−5.84, 6.47); *p* = 0.92]. Moderate heterogeneity was observed indicating variability across the studies (*I*^2^ = 50%, *p* = 0.16) (Supplementary Figure S5).

#### 3.5.2 Change in peak workload from baseline

**Figure 4:**
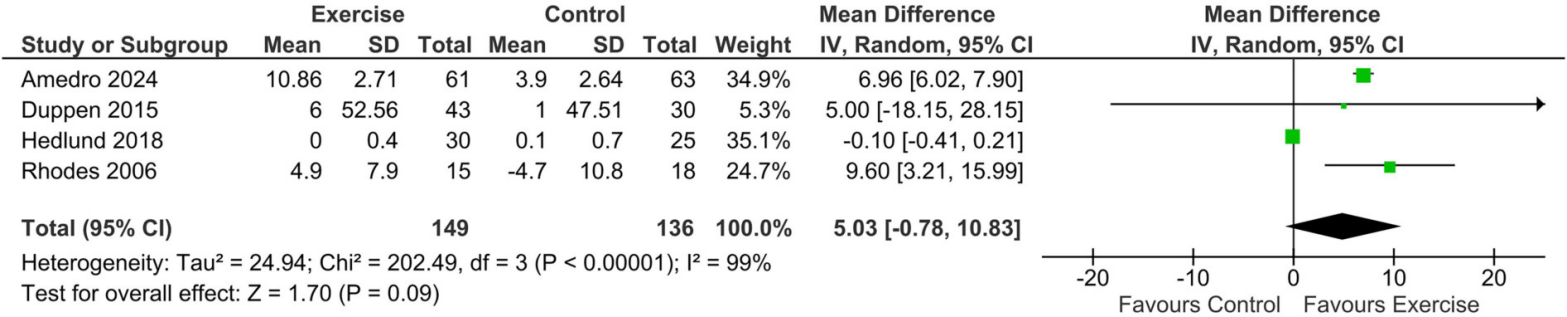
Forest plot illustrating the pooled mean difference (MD) for change in peak workload from baseline comparing exercise rehabilitation vs. standard care or no intervention. (Sensitivity analysis excluding Hedlund et al. is indicated.).

4 studies comprising 285 patients (exercise = 149, control = 136) assessed change in peak workload from baseline over 12 weeks. The analysis revealed no significant difference between exercise and control groups [MD = 5.03, 95% CI: (−0.78, 10.83); *p* = 0.09]. Substantial higher heterogeneity was observed indicating variability across the studies (*I*^2^ = 99%, *p* = 0.00001). The removal of Hedlund et al. resolved heterogeneity, resulting in a significant improvement in workload in the intervention arm (MD: 7.01; 95%CI: 6.08, 7.94; *P* < 0.0001) (Figure 4).

#### 3.5.3 Change in peak heart rate from baseline

4 studies 4 studies comprising 285 patients (exercise = 149, control = 136) assessed change in Peak heart rate from baseline over 12 weeks. The analysis revealed no significant difference between exercise and control groups [MD = 0.20, 95% CI: (−1.93,2.32); *p* = 0.86]. Low heterogeneity was observed indicating consistency across the studies (*I*^2^ = 15%, *p* = 0.32) (Supplementary Figure S6).

#### 3.5.4 Change in peak o2 pulse from baseline

3 studies comprising 230 patients (exercise = 119, control = 111) assessed change in peak o2 pulse from baseline over 12 weeks. The analysis revealed no significant difference between exercise and control groups [MD =0.43, 95% CI: (−1.41,2.28); *p* = 0.65]. Substantial higher heterogeneity was observed indicating variability across the studies (*I*^2^ = 72%, *p* = 0.03). Removal of Rhodes et al. significantly resolved the heterogeneity, and resulted in a significantly lower O2 pulse in the exercise group (MD: −0.18; 95%CI: −0.27, −0.09) (Supplementary Figure S7).

#### 3.5.5 Change in peak RER from baseline

3 studies comprising 161 patients (exercise = 88, control = 73) assessed change in peak RER from baseline over 12 weeks. The analysis revealed no significant difference between exercise and control groups [MD =0.02, 95% CI: (−0.01,0.04); *p* = 0.30]. Mild heterogeneity was observed indicating consistency across the studies (*I*^2^ = 30%, *p* = 0.24) (Supplementary Figure S8).

#### 3.5.6 Change in peak respiratory rate from baseline

2 studies comprising 88 patients (exercise = 45, control = 43) assessed change in peak respiratory rate from baseline over 12 weeks. The analysis revealed no significant difference between exercise and control groups [MD = 4.64 95% CI: (−7.28,16.57); *p* = 0.45]. Substantial higher heterogeneity was observed indicating variability across the studies (*I*^2^ = 82%, *p* = 0.02) (Supplementary Figure S9).

#### 3.5.7 Change in peak systolic blood pressure from baseline

2 studies comprising 212 patients (exercise = 106, control = 106) assessed change in peak systolic blood pressure from baseline over 12 weeks. The analysis revealed no significant difference between exercise and control groups [MD = 0.55 95% CI: (−0.36,1.46); *p* = 0.24] as shown in Table 2. No heterogeneity was observed indicating consistency across the studies (*I*^2^ = 0%, *p* = 0.56) (Supplementary Figure S10).

#### 3.5.8 Change in VAT % from baseline

2 studies comprising 157 patients (exercise = 76, control = 81) assessed change in VAT % from baseline over 12 weeks. The analysis revealed no significant difference between exercise and control groups [MD = 5.32 95% CI: (−5.21,15.68); *p* = 0.32]. Substantial higher heterogeneity was observed indicating variability across the studies (*I*^2^ = 89%, *p* = 0.002) (Supplementary Figure S11).

#### 3.5.9 Change in VE/VCO2 from baseline

4 studies comprising 289 patients (exercise = 152, control = 137) assessed change in VE/VCO2 from baseline over 12 weeks. The results revealed no statistically significant difference between exercise and control groups [MD = −0.63 95% CI: (−1.83,0.57); *p* = 0.30]. Moderate heterogeneity was observed across the studies (*I*^2^ = 35%, *p* = 0.20) (Supplementary Figure S12).

#### 3.5.10 Change in peak VO2% from baseline

2 studies comprising 157 patients (exercise = 76, control = 81) assessed change in peak VO2% from baseline over 12 weeks. The results revealed no statistically significant difference between exercise and control groups [MD = 4.98 95% CI: (−6.31,16.27); *p* = 0.39]. Substantial higher heterogeneity was observed indicating variability across the studies (*I*^2^ = 91%, *p* = 0.001) (Supplementary Figure S13). As compared to absolute VO2 peak (mL·kg⁻^1^·min⁻^1^) which quantifies the maximal oxygen uptake relative to body weight, reflecting an individual's cardiorespiratory fitness independent of age or sex. Peak VO2% is expressed as a percentage of predicted, therefore normalizes this value against population reference standards, enabling comparison across ages, sexes, and disease severities. Clinically, the normalized % predicted peak VO2 provides a more meaningful indicator of functional impairment and prognostic risk in pediatric CHD.

### 3.6 Certainty of evidence

The GRADE approach, using the GRADEpro Guideline Development Tool, was employed to assess the certainty of evidence. A detailed assessment is shown in Supplementary Table S2.

## 4 Discussion

Our analysis concluded that there was no statistically significant improvement in the hemodynamic and cardiac profile following intervention with rehabilitative therapies in children with CHDs. While rehabilitation improved duration in exercise significantly on follow-up, change in other cardiometabolic parameters like oxygen consumption, peak heart rate, workload, respiratory rate and blood pressure remained similar in both groups. Peak workload improved following exercise among children with CHDs other than Fontan physiology.

Rehabilitative interventions and exercise are generally considered to be safe in children with CHDs (30). Guidelines necessitate equal duration of exercise as their healthy counterparts, recommending 60 min of moderate-to-vigorous exercise (31). We recorded a significantly higher endurance in the form of longer duration of exercise among children receiving any rehabilitative intervention. Similar conclusions were drawn by Callaghan et al., who noted a significant improvement in exercise tolerance (25). Previous studies, such as those by Keteyian et al., have similarly demonstrated that exercise training increases exercise capacity in heart failure patients, likely due to peripheral adaptations (e.g., enhanced skeletal muscle oxidative capacity) rather than central cardiac improvements alone (32). However, the improvement demonstrated might be too limited; future studies should assess whether this translates to meaningful functional benefits in daily life.

Further, we noted no improvement in peak VO2 between the two groups during stress tests. This implies exercise rehabilitation may not have improved respiratory reserve significantly. The insignificance persisted despite the removal of any study, while heterogeneity was in-resolvable. This may be due to the different follow-up periods across different groups, along with the variability in patient population. The pre-existing activity of the child may play a role as well; children who may have already been exercising regularly may not have demonstrated any significant benefit over and above their pre-existing baseline conditions. The variability may stem from differences in CHD severity, exercise modality, or the length and intensity of intervention protocols. Our findings was consistent with a Cochrane systematic review by Wadey et al. (33), pooled data revealed a mean difference in peak VO₂ of ∼1.9 ml·kg⁻^1^·min⁻^1^ (95% CI −0.22 to 3.99) favouring intervention vs. control (moderate-certainty evidence) and a mean improvement in submaximal cardiorespiratory fitness (CRF) of ∼2.05 ml·kg⁻^1^·min⁻^1^ (95% CI 0.05–4.05) (moderate-certainty). Individuals with CHD appear to lead to small but measurable improvements in CRF and muscular strength, and modest increases in daily physical activity, with no serious adverse events reported.

No significant difference was noted in peak workload in our analysis. For peak workload, heterogeneity was resolved on removal of Hedlund et al., which resulted in a significantly improved workload. Hedlund et al. analyzed children with Fontan physiology only, suggesting that exercise may not have a significant role in improving parameters among patients with this disease (21).

Our analysis also noted no significant improvement in peak heart rate. Given the chronotropic incompetence often seen in CHD—especially post-Fontan or in those on beta-blockers—exercise-induced modulation of peak heart rate may be blunted (34). This suggests that heart rate metrics alone may be suboptimal markers of training response in this cohort.

No change was noted in peak systolic blood pressure across both groups. The lack of change is consistent with prior observations that blood pressure responses in pediatric CHD populations are tightly autoregulated unless impaired ventricular function exists (35).

While a clinically significant improvement in exercise duration was noted, most cardiopulmonary parameters showed no statistically significant differences between exercise and control groups. However, this does not negate the potential benefits of physical training in pediatric CHD populations. Subgroup-specific improvements and the high variability suggest that outcomes may depend heavily on the underlying defect type, surgical status, baseline fitness, and exercise protocol. Given the high heterogeneity in several outcomes, standardized, multicenter randomized trials are urgently needed, stratifying participants by CHD subtype and tailoring intervention intensity accordingly. Further, long-term follow-up is essential to assess whether early changes in exercise capacity translate to improved morbidity, quality of life, and survival in adulthood.

Despite the insignificance noted in our analysis, individualized and structured aerobic exercise prescriptions have emerged as critical adjuncts in the management of pediatric patients with CHD, demonstrating clinically significant benefits across both cyanotic and non-cyanotic phenotypes (22, 28). This may be due to differences across exercise administered. Some exercise parameters may not have demonstrated statistically significant changes due to limitations in their sensitivity to capture early or subtle functional improvements, especially in pediatric populations. For instance, measures like peak VO2 or peak heart rate may not reflect peripheral muscular adaptations or psychosocial benefits resulting from exercise training (30). Moreover, chronotropic incompetence, prevalent in certain CHD subtypes or post-surgical states, may inherently limit heart rate responsiveness during exertion, thus masking improvements in cardiovascular efficiency (33). The duration of follow-up in most studies was relatively short (typically under 6 months), which may not have been sufficient to detect structural or physiological changes that evolve over longer periods. Additionally, variability in test protocols, inconsistent definitions of outcome measures, and potential ceiling effects in children who were already physically active at baseline could further dilute observable effects.

Thus, while statistical differences were not detected in several cardiopulmonary parameters, these findings do not necessarily negate the presence of meaningful clinical or functional benefits. Evidence underscores the need for precision-based exercise dosing, integrating baseline cardiopulmonary assessment, residual lesion status, and chronotropic competence. In a mediation analysis of adolescents with complex CHD, higher exercise capacity (as measured by peak VO₂) fully mediated the positive relationship between physical activity and both self- and parent-reported health-related quality of life (HRQoL). These findings suggest that structured aerobic training may enhance psychosocial and functional domains of HRQoL primarily via improved cardiorespiratory fitness (36).

Ultimately, such exercise paradigms contribute to improved quality of life, reduced hospitalization, and long-term cardiovascular resilience in pediatric CHD populations. Programs should ideally be supervised, multidisciplinary, and personalized, taking into account growth, developmental milestones, and psychological well-being. Incorporating motivational interviewing, family engagement, and school-based physical activity programs may improve adherence and optimize benefits.

## 5 Limitations

This study is restricted by several limitations. First, the small number of studies (*n* = 10) included, and their small sample sizes restrict statistical power and generalizability. Second, high heterogeneity in most outcomes, especially peak VO2 and workload, indicated differences and variability in study design, CHD subtypes, and intervention protocols, obscuring the synthesis of results. Third, the consideration of both RCTs and observational studies brings potential bias, as observational studies are more prone to interfering. Fourth, the insufficiency of standardized exercise protocols across studies obstructs direct comparisons and the advancement of specific guidelines. Finally, the short follow-up time in most studies prevents evaluation of long-term outcomes, such as morbidity, mortality, or sustained quality of life enhancements.

## 6 Future directions

Future research should emphasize on large-scale, multicenter randomized controlled trials to tackle the current evidence gaps. These trials should categorize participants by CHD subtype (e.g., Fontan vs. tetralogy of Fallot) and adapt exercise intensity and modality to personalized patient characteristics. Uniform treatment protocols, comprising duration, frequency, and type of exercise (e.g., aerobic vs. resistance training), are necessary to decrease heterogeneity and improve comparability. Adoption of pediatric cardiopulmonary exercise testing Z-scores (CPET Z-scores) in future analyses could reduce inter-study heterogeneity and enhance comparability without necessarily changing the direction of results. It also helps overcome the limitations of %predicted values based on linear models that are not reliable for extreme weight children (37).

Long-term follow-up studies are necessary to assess whether early enhancements in exercise capacity interpret to reduced morbidity, enhanced quality of life, and improved survival in adulthood. Additionally, including patient-centered outcomes, such as health-related quality of life and psychosocial quality, could contribute to a more holistic evaluation of exercise benefits. Multidisciplinary team approaches incorporating motivational interviewing, family involvement, and school-based physical activity efforts may enhance compliance and optimize outcomes. Finally, evaluating the role of wearable technology and tele-rehabilitation could improve the accessibility and personalization of exercise interventions for pediatric CHD patients.

## 7 Conclusion

This meta-analysis shows that exercise-based rehabilitation programs notably improve exercise duration in children with congenital heart defects (CHD), but they do not provide statistically considerable improvements in most cardiopulmonary parameters, such as peak VO2, workload, heart rate, or blood pressure. The noted increase in exercise duration indicated potential for improved physical endurance, but the absence of constant improvements across other evaluated outcomes reflects the difficulty of utilizing exercise interventions in this population. High heterogeneity among studies, likely caused by differences in CHD subtypes, baseline fitness, and intervention strategies, highlights the need for more standardized, multicenter randomized controlled trials. Future research should aim on adapting exercise programs to specific CHD subtypes, maximizing intervention intensity, and assessing long-term effects on morbidity, quality of life, and survival. Monitored, multidisciplinary, and personalized rehabilitation programs, including family and school-based engagement, are suggested to increase adherence and clinical advantages in pediatric CHD patients.

## References

[1] Awosika A Hillman AR Millis RM Adeniyi MJ. Cardiac rehabilitation and cardiopulmonary fitness in children and young adults with congenital heart diseases: a critically appraised topic. Cureus. (2022) 14(11):e31483. 10.7759/cureus.31483 36408315 PMC9665330

[2] Centers for Disease Control and Prevention (CDC). Hospital stays, hospital charges, and in-hospital deaths among infants with selected birth defects—united States, 2003. MMWR Morb Mortal Wkly Rep*.* (2007) 56:25–9. 17230142

[3] Wang H Naghavi M Allen C Barber RM, GBD 2015 Mortality and Causes of Death Collaborators. Global, regional, and national life expectancy, all-cause mortality, and cause-specific mortality for 249 causes of death, 1980–2015: a systematic analysis for the global burden of disease study 2015. Lancet. (2016) 388(10053):1459–544. 10.1016/S0140-6736(16)31012-1 Erratum in: Lancet. 2017 January 7;389(10064):e1. doi: 10.1016/S0140-6736(16)32605-8 27733281 PMC5388903

[4] Tchervenkov CI Jacobs JP Bernier PL Stellin G Kurosawa H Mavroudis C The improvement of care for paediatric and congenital cardiac disease across the world: a challenge for the world society for pediatric and congenital heart surgery. Cardiol Young. (2008) 18(Suppl 2):63–9. 10.1017/S1047951108002801 19063776

[5] Amiard V Jullien H Nassif D Bach V Maingourd Y Ahmaidi S. Effects of home-based training at dyspnea threshold in children surgically repaired for congenital heart disease. Congenit Heart Dis. (2008) 3(3):191–9. 10.1111/j.1747-0803.2008.00191.x 18557882

[6] Böhm B Oberhoffer R. Vascular health determinants in children. Cardiovasc Diagn Ther. (2019) 9(Suppl 2):S269–80. 10.21037/cdt.2018.09.16 31737535 PMC6837937

[7] McCrindle BW Williams RV Mital S Clark BJ Russell JL Klein G Physical activity levels in children and adolescents are reduced after the fontan procedure, independent of exercise capacity, and are associated with lower perceived general health. Arch Dis Child. (2007) 92(6):509–14. 10.1136/adc.2006.105239 PMID: 17307794; PMCID: PMC2066169. 17307794 PMC2066169

[8] Dulfer K Helbing WA Duppen N Utens EM. Associations between exercise capacity, physical activity, and psychosocial functioning in children with congenital heart disease: a systematic review. Eur J Prev Cardiol. (2014) 21(10):1200–15. 10.1177/2047487313494030 23787793

[9] Strong WB Malina RM Blimkie CJ Daniels SR Dishman RK Gutin B Evidence based physical activity for school-age youth. J Pediatr. (2005) 146(6):732–7. 10.1016/j.jpeds.2005.01.055 PMID: 15973308. 15973308

[10] Ortega FB Ruiz JR Castillo MJ Sjöström M. Physical fitness in childhood and adolescence: a powerful marker of health. Int J Obes (Lond). (2008) 32(1):1–11. 10.1038/sj.ijo.0803774 18043605

[11] Bradley LM Galioto FM Jr Vaccaro P Hansen DA Vaccaro J. Effect of intense aerobic training on exercise performance in children after surgical repair of tetralogy of fallot or complete transposition of the great arteries. Am J Cardiol. (1985) 56(12):816–8. 10.1016/0002-9149(85)91155-5 4061316

[12] Lisón JF Real-Montes JM Torró I Arguisuelas MD Alvarez-Pitti J Martínez-Gramage J Exercise intervention in childhood obesity: a randomized controlled trial comparing hospital-versus home-based groups. Acad Pediatr. (2012) 12(4):319–25. 10.1016/j.acap.2012.03.003 PMID: 22634075. 22634075

[13] Dold SK Haas NA Apitz C. Effects of sports, exercise training, and physical activity in children with congenital heart disease-A review of the published evidence. Children (Basel). (2023) 10(2):296. 10.3390/children10020296 36832425 PMC9955038

[14] Page MJ McKenzie JE Bossuyt PM Boutron I Hoffmann TC Mulrow CD The PRISMA 2020 statement: an updated guideline for reporting systematic reviews. Br Med J. (2021) 372:n71. 10.1136/bmj.n71 PMID: 33782057; PMCID: PMC8005924. 33782057 PMC8005924

[15] Sterne JAC Savović J Page MJ Elbers RG Blencowe NS Boutron I Rob 2: a revised tool for assessing risk of bias in randomised trials. Br Med J. (2019) 366:l4898. 10.1136/bmj.l4898 PMID: 31462531. 31462531

[16] Schünemann HJ Cuello C Akl EA Mustafa RA Meerpohl JJ Thayer K GRADE Guidelines: 18. How ROBINS-I and other tools to assess risk of bias in nonrandomized studies should be used to rate the certainty of a body of evidence. J Clin Epidemiol. (2019) 111:105–14. 10.1016/j.jclinepi.2018.01.012 PMID: 29432858; PMCID: PMC6692166. 29432858 PMC6692166

[17] Santesso N Carrasco-Labra A Langendam M Brignardello-Petersen R Mustafa RA Heus P Improving GRADE evidence tables part 3: detailed guidance for explanatory footnotes supports creating and understanding GRADE certainty in the evidence judgments. J Clin Epidemiol. (2016) 74:28–39. 10.1016/j.jclinepi.2015.12.006 PMID: 26796947. 26796947

[18] Shao SC Kuo LT Huang YT Lai PC Chi CC. Using grading of recommendations assessment, development, and evaluation (GRADE) to rate the certainty of evidence of study outcomes from systematic reviews: a quick tutorial. Dermatol Sin. (2023) 41(1):3. 10.4103/ds.DS-D-22-00154

[19] Higgins JP Thompson SG Deeks JJ Altman DG. Measuring inconsistency in meta-analyses. Br Med J. (2003) 327(7414):557–60. 10.1136/bmj.327.7414.557 12958120 PMC192859

[20] DerSimonian R Laird N. Meta-analysis in clinical trials. Control Clin Trials. (1986) 7(3):177–88. 10.1016/0197-2456(86)90046-2 3802833

[21] Hedlund ER Lundell B Villard L Sjöberg G. Reduced physical exercise and health-related quality of life after Fontan palliation. Acta Paediatr. (2016) 105(11):1322–8. 10.1111/apa.13544 27515293

[22] Moalla W Elloumi M Chamari K Dupont G Maingourd Y Tabka Z Training effects on peripheral muscle oxygenation and performance in children with congenital heart diseases. Appl Physiol Nutr Metab. (2012) 37(4):621–30. 10.1139/h2012-036 PMID: 22554184. 22554184

[23] Amedro P Gavotto A Huguet H Souilla L Huby AC Matecki S Early hybrid cardiac rehabilitation in congenital heart disease: the QUALIREHAB trial. Eur Heart J. (2024) 45(16):1458–73. 10.1093/eurheartj/ehae085 PMID: 38430485; PMCID: PMC11032713. 38430485 PMC11032713

[24] Rhodes J Curran TJ Camil L Rabideau N Fulton DR Gauthier NS Sustained effects of cardiac rehabilitation in children with serious congenital heart disease. Pediatrics. (2006) 118(3):e586–93. 10.1542/peds.2006-0264 PMID: 16950950. 16950950

[25] Callaghan S Morrison ML McKeown PP Tennyson C Sands AJ McCrossan B Exercise prescription improves exercise tolerance in young children with CHD: a randomised clinical trial. Open Heart. (2021) 8(1):e001599. 10.1136/openhrt-2021-001599 PMID: 33990433; PMCID: PMC8127973. 33990433 PMC8127973

[26] Jacobsen RM Ginde S Mussatto K Neubauer J Earing M Danduran M. Can a home-based cardiac physical activity program improve the physical function quality of life in children with Fontan circulation? Congenit Heart Dis. (2016) 11(2):175–82. 10.1111/chd.12330 26879633

[27] Klausen SH Andersen LL Søndergaard L Jakobsen JC Zoffmann V Dideriksen K Effects of eHealth physical activity encouragement in adolescents with complex congenital heart disease: the PReVaiL randomized clinical trial. Int J Cardiol. (2016) 221:1100–6. 10.1016/j.ijcard.2016.07.092 PMID: 27448540. 27448540

[28] Fredriksen PM Ingjer E Thaulow E. Physical activity in children and adolescents with congenital heart disease. Aspects of measurements with an activity monitor. Cardiol Young. (2000) 10(2):98–106. 10.1017/s1047951100006545 10817292

[29] Duppen N Etnel JR Spaans L Takken T van den Berg-Emons RJ Boersma E Does exercise training improve cardiopulmonary fitness and daily physical activity in children and young adults with corrected tetralogy of fallot or fontan circulation? A randomized controlled trial. Am Heart J. (2015) 170(3):606–14. 10.1016/j.ahj.2015.06.018 PMID: 26385046. 26385046

[30] Selamet Tierney ES. The benefit of exercise in children with congenital heart disease. Curr Opin Pediatr. (2020) 32(5):626–32. 10.1097/MOP.0000000000000942 32868597

[31] van Deutekom AW Lewandowski AJ. Physical activity modification in youth with congenital heart disease: a comprehensive narrative review. Pediatr Res. (2021) 89(7):1650–8. 10.1038/s41390-020-01194-8 33049756 PMC8249230

[32] Keteyian SJ Hibner BA Bronsteen K Kerrigan D Aldred HA Reasons LM Greater improvement in cardiorespiratory fitness using higher-intensity interval training in the standard cardiac rehabilitation setting. J Cardiopulm Rehabil Prev. (2014) 34(2):98–105. 10.1097/HCR.0000000000000049 PMID: 24531203. 24531203

[33] Wadey CA Pieles G Stuart G Taylor R Long L Williams CA. Cochrane corner: physical activity interventions for people with congenital heart disease. Heart. (2021) 107:447–9. 10.1136/heartjnl-2020-318459 PMID: 33452120. 33452120

[34] Norozi K Wessel A Alpers V Arnhold JO Binder L Geyer S Chronotropic incompetence in adolescents and adults with congenital heart disease after cardiac surgery. J Card Fail. (2007) 13(4):263–8. 10.1016/j.cardfail.2006.12.002 PMID: 17517345. 17517345

[35] Brady KM Mytar JO Lee JK Cameron DE Vricella LA Thompson WR Monitoring cerebral blood flow pressure autoregulation in pediatric patients during cardiac surgery. Stroke. (2010) 41(9):1957–62. 10.1161/STROKEAHA.109.575167 PMID: 20651273; PMCID: PMC5498798. 20651273 PMC5498798

[36] Kim HJ Jae SY Choo J Yoon JK Kim SH Königstein K Mediating effects of exercise capacity on the association between physical activity and health-related quality of life among adolescents with complex congenital heart disease. Am J Hum Biol. (2019) 31(6):e23297. 10.1002/ajhb.23297 PMID: 31321831. 31321831

[37] Gavotto A Mura T Rhodes J Yin SM Hager A Hock J Reference values of aerobic fitness in the contemporary paediatric population. Eur J Prev Cardiol. (2023) 30(9):820–9. 10.1093/eurjpc/zwad054 36809338

