# Supplementary material for: Impact of individualized and structured aerobic exercise on clinical outcomes in pediatric congenital heart diseases with post-surgical rehabilitation: a meta-analysis
Source: Front Surg. 2025 Nov 26;12:1622547. doi: 10.3389/fsurg.2025.1622547 (PMC12689524; doi:10.3389/fsurg.2025.1622547)
Supplement: Supplementary file 1 [file Datasheet1.docx]

# Supplementary Table S1. Detailed Search Strategy

| Database | Search Terms | Filters/Limits Applied | Date of Search | Results Retrieved |
| --- | --- | --- | --- | --- |
| PubMed/MEDLINE | ("Heart Defects, Congenital"[MeSH Terms] OR "Long QT Syndrome"[MeSH Terms] OR "congenital heart disease"[Title/Abstract] OR "congenital heart defect"[Title/Abstract] OR "pediatric heart disease"[Title/Abstract] OR "Tetralogy of Fallot"[Title/Abstract] OR "Fontan"[Title/Abstract] OR "long QT syndrome"[Title/Abstract])  AND  ("Exercise"[MeSH Terms] OR "Exercise Therapy"[MeSH Terms] OR "Cardiac Rehabilitation"[MeSH Terms] OR "exercise training"[Title/Abstract] OR "aerobic exercise"[Title/Abstract] OR "cardiopulmonary rehabilitation"[Title/Abstract] OR "physical activity"[Title/Abstract])  AND  (individualized[Title/Abstract] OR tailored[Title/Abstract] OR personalized[Title/Abstract] OR "cardiopulmonary exercise testing"[Title/Abstract] OR CPET[Title/Abstract] OR "heart rate"[Title/Abstract])  AND  ("Randomized Controlled Trial"[Publication Type] OR "randomized controlled trial"[Title/Abstract] OR RCT[Title/Abstract])  AND  ("Child"[MeSH Terms] OR "Adolescent"[MeSH Terms] OR pediatric[Title/Abstract] OR child*[Title/Abstract] OR adolescent*[Title/Abstract])  AND  ("Oxygen Consumption"[MeSH Terms] OR "VO2 max"[Title/Abstract] OR "VO2 peak"[Title/Abstract] OR "cardiopulmonary fitness"[Title/Abstract] OR "quality of life"[Title/Abstract] OR "functional capacity"[Title/Abstract] OR "psychological well-being"[Title/Abstract] OR "cardiovascular risk"[Title/Abstract])  AND  ("2000/01/01"[PDAT] : "2025/12/31"[PDAT]) | English, Jan 2000 – Dec 2025 | 23 April 2025 | 11 |
| Embase | ('congenital heart disease'/exp OR 'long qt syndrome'/exp OR 'congenital heart disease':ti,ab OR 'congenital heart defect':ti,ab OR 'pediatric heart disease':ti,ab OR 'tetralogy of fallot':ti,ab OR 'fontan':ti,ab OR 'long qt syndrome':ti,ab) AND ('exercise'/exp OR 'exercise therapy'/exp OR 'cardiac rehabilitation'/exp OR 'exercise training':ti,ab OR 'aerobic exercise':ti,ab OR 'cardiopulmonary rehabilitation':ti,ab OR 'physical activity':ti,ab) AND (individualized:ti,ab OR tailored:ti,ab OR personalized:ti,ab OR 'cardiopulmonary exercise testing':ti,ab OR cpet:ti,ab OR 'heart rate':ti,ab) AND ('randomized controlled trial'/exp OR 'randomized controlled trial':ti,ab OR rct:ti,ab) AND ('child'/exp OR 'adolescent'/exp OR pediatric:ti,ab OR child*:ti,ab OR adolescent*:ti,ab) AND ('oxygen consumption'/exp OR 'vo2 max':ti,ab OR 'vo2 peak':ti,ab OR 'cardiopulmonary fitness':ti,ab OR 'quality of life':ti,ab OR 'functional capacity':ti,ab OR 'psychological well-being':ti,ab OR 'cardiovascular risk':ti,ab) | English, Jan 2000 – Dec 2025 | 23 April 2025 | 23 |
| Web of Science | ('congenital heart disease'/exp OR 'long qt syndrome'/exp OR 'congenital heart disease':ti,ab OR 'congenital heart defect':ti,ab OR 'pediatric heart disease':ti,ab OR 'tetralogy of fallot':ti,ab OR 'fontan':ti,ab OR 'long qt syndrome':ti,ab) AND ('exercise'/exp OR 'exercise therapy'/exp OR 'cardiac rehabilitation'/exp OR 'exercise training':ti,ab OR 'aerobic exercise':ti,ab OR 'cardiopulmonary rehabilitation':ti,ab OR 'physical activity':ti,ab) AND (individualized:ti,ab OR tailored:ti,ab OR personalized:ti,ab OR 'cardiopulmonary exercise testing':ti,ab OR cpet:ti,ab OR 'heart rate':ti,ab) AND ('randomized controlled trial'/exp OR 'randomized controlled trial':ti,ab OR rct:ti,ab) AND ('child'/exp OR 'adolescent'/exp OR pediatric:ti,ab OR child*:ti,ab OR adolescent*:ti,ab) AND ('oxygen consumption'/exp OR 'vo2 max':ti,ab OR 'vo2 peak':ti,ab OR 'cardiopulmonary fitness':ti,ab OR 'quality of life':ti,ab OR 'functional capacity':ti,ab OR 'psychological well-being':ti,ab OR 'cardiovascular risk':ti,ab) | English, Jan 2000 – Dec 2025 | 23 April 2025 | 6 |

Supplementary Table S2 : Grade assesment


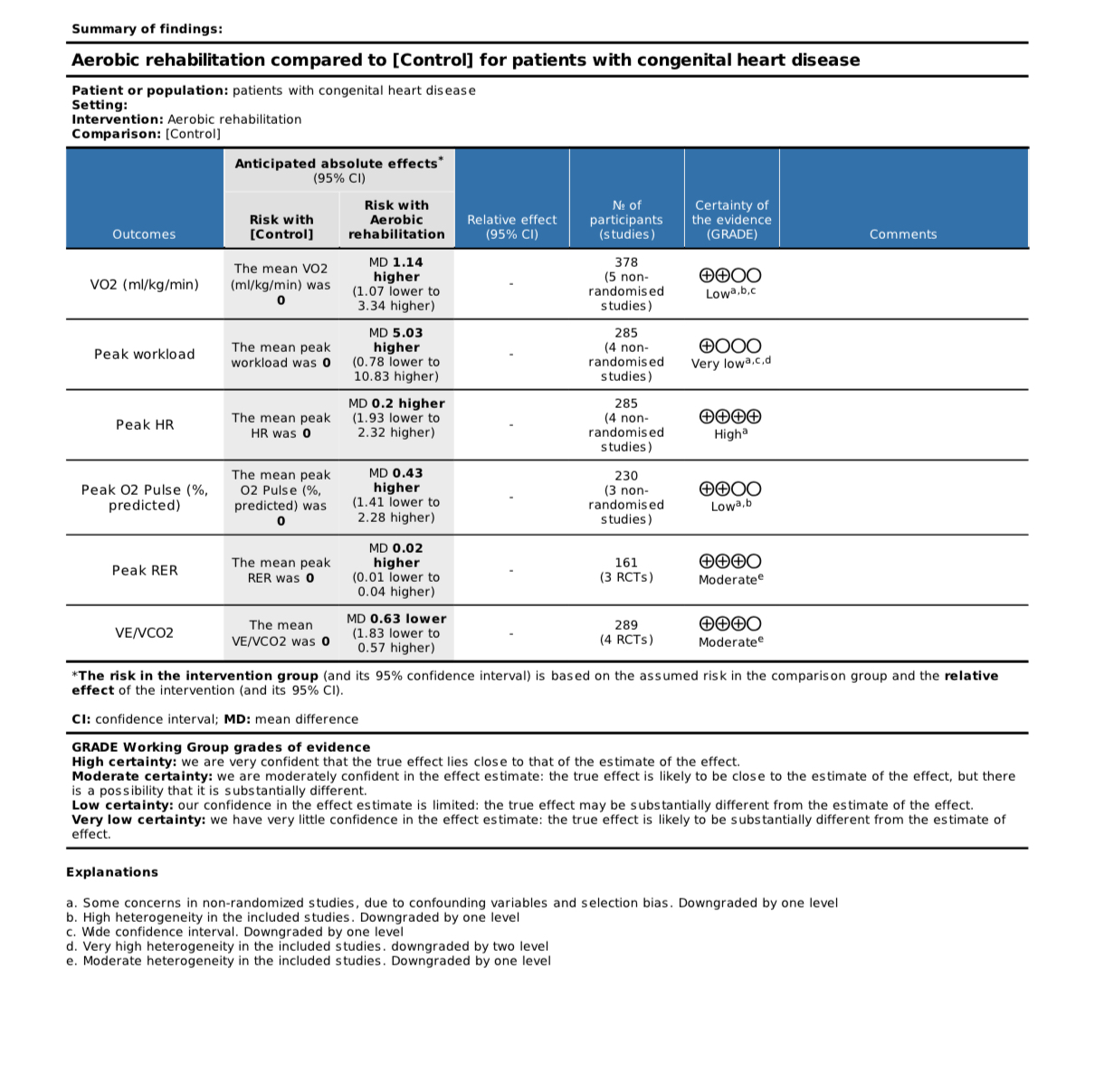


Supplementary Figures

### Figure S1: RoB 2 Traffic-Light Plot (RCTs)

*
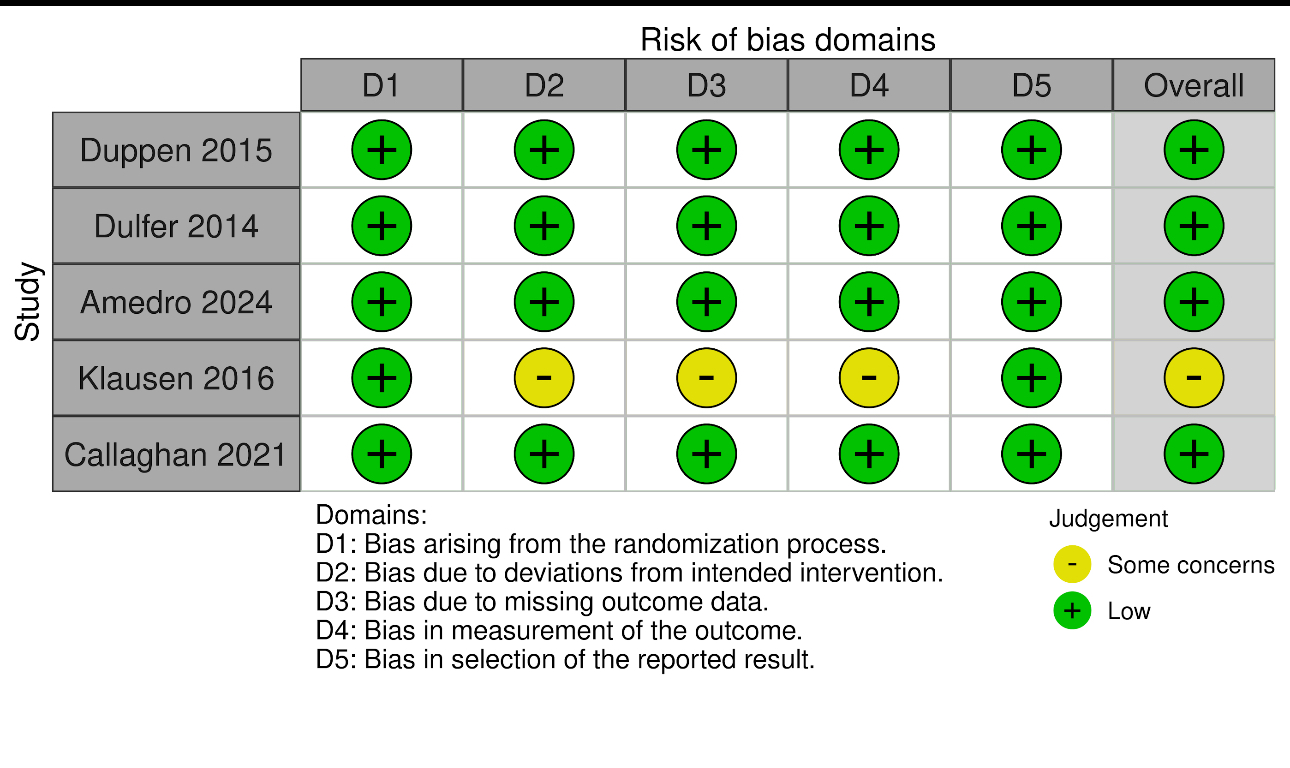
*

**Figure S2: RoB 2 Summary Bar Plot (RCTs)**


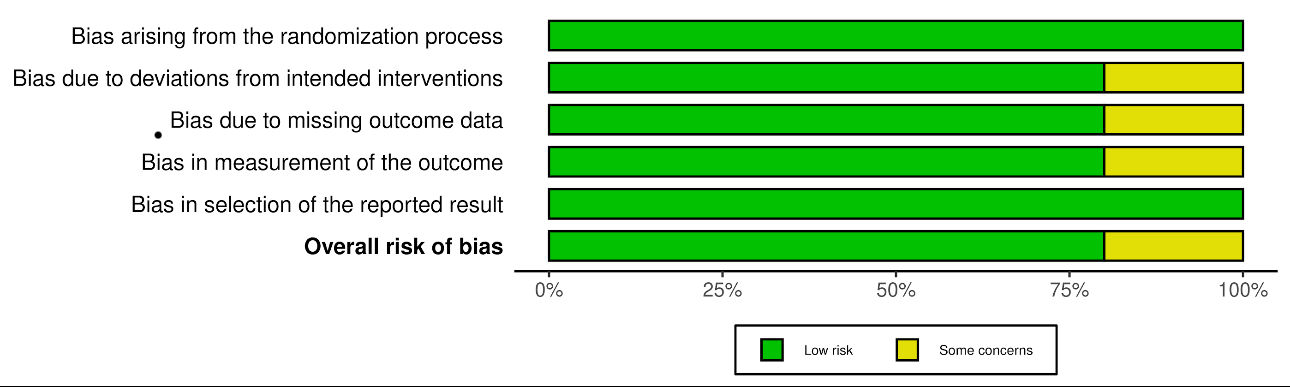


**Figure S3: ROBINS-I Summary Bar Plot (Observational Studies)**


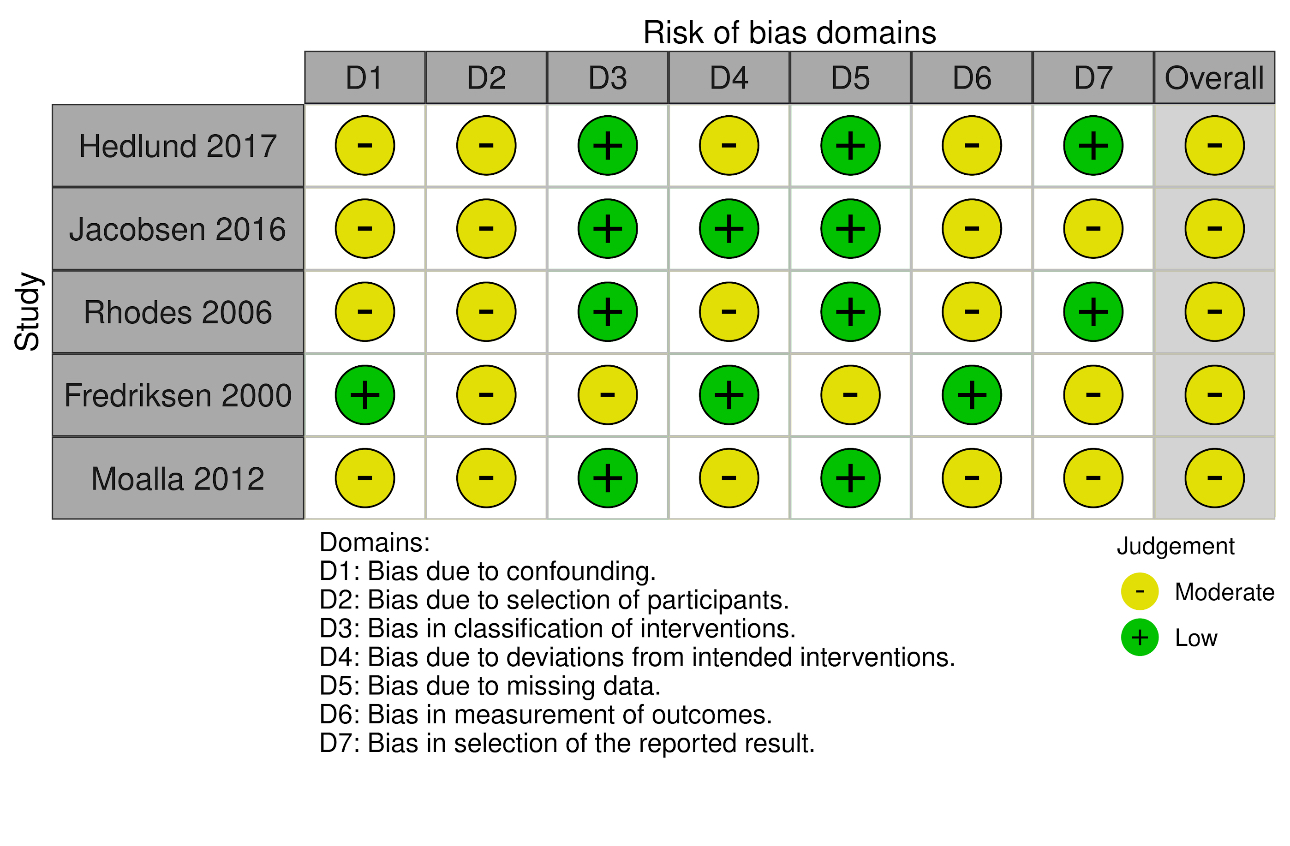


**Figure S4: ROBINS-I Traffic-Light Plot (Observational Studies)**

**
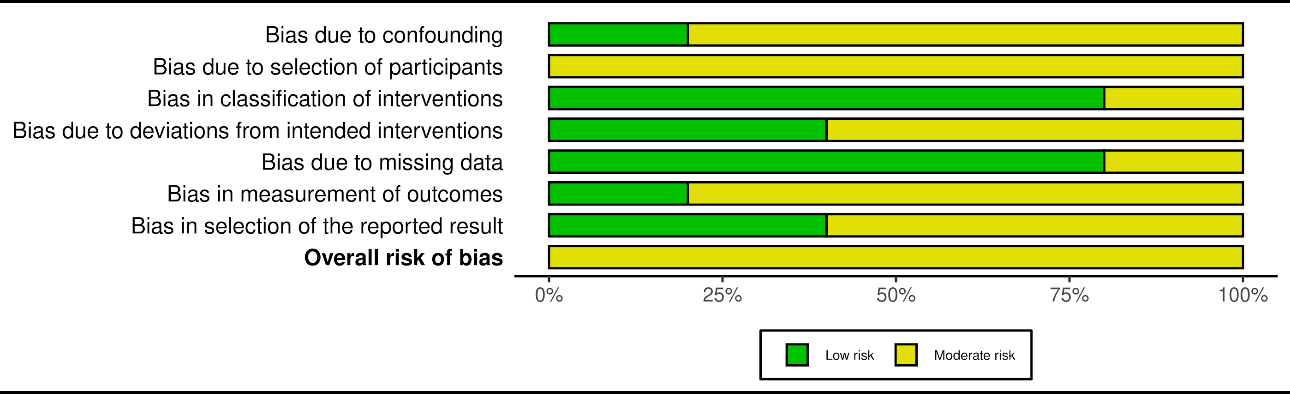
**

**Figure S5: Pooled MD in Moderate/Severe Exercise capacity**

**
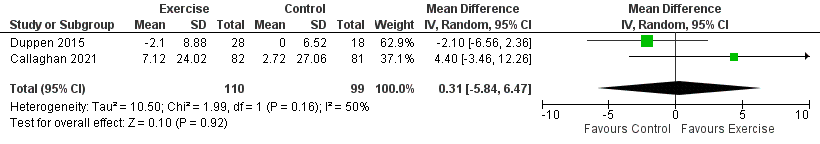
**

**Figure S6: Pooled MD in Peak Heart Rate**

**
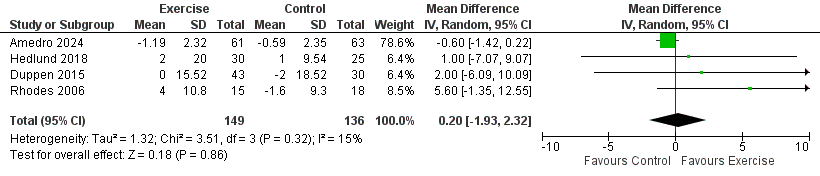
**

**Figure S7:** **Pooled MD in Peak O₂ Pulse**

**
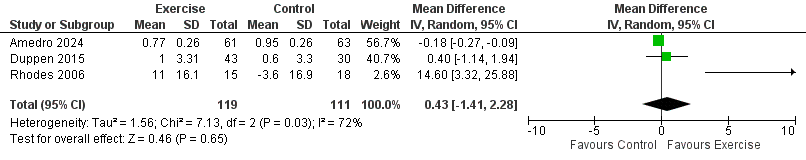
**

**Figure S8: Pooled MD in Peak RER**

**
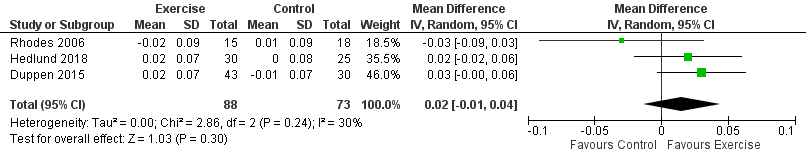
**

**Figure S9: Pooled MD in Peak Respiratory Rate**

**
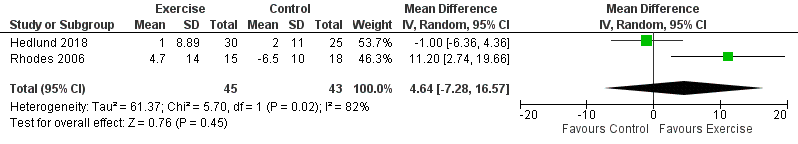
**

**Figure S10:Pooled MD in Peak SBP**
